# Books and Authors

**Published:** 1906-11

**Authors:** 


### BOOKS AND AUTHORS.

**The Practice of Gynecology,** in original contributions by American authors, edited by **J. Wesley Bovée,** M.D., Professor of Gynecology in George Washington University, Washington, D. C. In one octavo volume, containing 838 pages, with 382 engravings and 60 full page plates in colors and mono-chrome. Lea Brothers & Company, Philadelphia and New York. 1906. (Cloth, $6.00, leather, $7.00, half morocco, $8.00, net).

The name of this work has been well chosen. "The Practice of Gynecology" is a term that properly applies to it and is much better than other somewhat worn expressions such as "textbook," "treatise," and the like. Moreover, it is of broader scope than textbook or treatise in general, since it includes in its domain not only a discussion of the diseases of the generative organs of woman, but also maladies of the urinary system and rectum. Furthermore, it is one of a series intended to cover the fields of gynecology, obstetrics, and pediatrics, to be known as the Practitioner's Library.

The work is encyclopedic in character, the contributors being seven in number, including the editor. Dr. Bovée has won a place for himself among the foremost gynecologists of the country, and also has demonstrated his ability as a writer through the numerous contributions he has made to gynecology, medical and surgical, during the past decade.. In the selection of his coadjutors he has shown wise discrimination, all being men of distinction in the branch of medicine they have chosen as a specialty, some, indeed, being conspicuously eminent.

The examination of the pelvic contents forms the subject of the opening chapter, written by Dr. X. O. Werder, of Pittsburg, who is one of the most accomplished of the younger middle-aged men. He is an operator of great skill, and his judgment is superb. He speaks of what he knows to be correct from personal experience. He also writes the chapter on the technic of abdominal operations, as well as the one on extrauterine pregnancy. The three contributions of Dr. Werder could scarcely be excelled in sound teaching, practical value, or conciseness in expression. Dr. Bovée, the editor, contributes the second chapter, the subject of which is the develomental anomalies of the female generative organs, which is elaborately illustrated. He also contributes six other chapters, the titles of which are sterility, diseases of the rectum and anus, abnormal conditions of the urinary tract and surgical conditions of the kidney, surgical conditions of the ureter, affections of the bladder, and the final chapter, which deals with affections of the urethra. These topics all are important and have been presented with scientific skill.

Dr. J. Riddle Goffe, of New York, contributes chapters with titles as follows: menstruation, displacements of the uterus, the vaginal method of operating, and the after-treatment and complications of abdominal operations. Dr. George H. Noble contributes chapters on fecal fistulæ connecting with the female generative organs, urinary fistulæ connecting with the female generative organs, lacerations of the perineum, and diseases and injuries of the vulva and vagina. The subjects of the chapters contributed by Dr. G. Brown Miller, of Washington, are inflammation of the uterus, lacerations of the cervix and subinvolution and hyperinvolution of the uterus, inversion of the uterus, fibromata of the uterus, including their treatment, and malignant tumors of the uterus.

The anatomy of the fallopian tubes and ovaries, diseases of the tubes, and extrauterine pregnancy form the subject of one chapter, and diseases of the ovary the topic of another, written by Dr. Benjamin R. Schenck, of Detroit, while three chapters on the infections of the tubes and ovaries are presented by Dr. Thomas J. Watkins, of Chicago. It will be observed from this exhibit of the subjects dealt with by the several contributors that every essential in the domain of gynecology is covered, and that, too, by men amply qualified to discuss the topics which they have been chosen to handle.

In summing up we must not fail to mention the illustrations, many of which are original, some are superb, and all are excellent, giving a graphic description of the text that is always appreciated by the careful reader. Finally, the book by and large is a most gratifying exposition of the present status of gynecology, which cannot fail to meet the wants of students and practitioners of medicine.

---

**Reference Handbook of the Diseases of Children.** For Students and Practitioners. By **Professor Ferdinand Frühwald,** of Vienna. Edited, with additions, by **Thompson S. Westcott,** M.D., Associate Professor of Diseases of Children in the University of Pennsylvania. Octavo, 553 pages, with 176 illustrations. Philadelphia and London: W. B. Saunders Company. 1906. (Cloth, $4.50 net; Half Morocco, $5.50 net).

The author of this treatise had been engaged in teaching more than twenty years before he attempted to bring out a manual on the subject of pediatrics. Professor Frühwald experienced the lack of a compact teaching manual, hence set to work to supply one. Those who are familiar with the book in the original German have given favorable indorsement of it and now the American profession is given an opportunity to pass judgment upon its

The alphabetical classification has been followed, as affording the most practical form for a reference book; important pathologic findings have been but lightly referred to; but symptomatology has been dealt with comprehensively, thus opening the way to more assured diagnosis. In the line of treatment only the most approved measures are adopted, while the most recent achievements in therapeutics are described at length. Due attention is given, of course, to prophylactic, hygienic, and dietetic procedures in relation to each disease while it is under consideration.

Frühwald is a believer in the use of pictorial representation as an aid to the recognition and understanding of many diseases and, therefore, has taken pains to introduce illustrations of a considerable number of the rarer conditions met with in practice. These are made from photographs, sketches from nature, or diagramatic drawings, nearly all of which are taken from his own clinic at the Vienna Poliklinik. It is of interest to note that, by special arrangement with the foreign publisher, Mr. Franz Deuticke, of Leipsig and Vienna, the illustrations in this edition are, for the most part, printed from the original plates used in the German edition, which insures accuracy. A few extra plates from accredited American sources are added,—all of which testify to the enterprise of the American editor and the publishers.

It is evident, as a concluding remark, that this reference handbook becomes at once a distinct addition to the literary armament of the student, for whom it is particularly intended.

---

**The Practical Medicine Series of Year Books.** Ten volumes. Series of 1906. Issued under the general editorial charge of **Gustavus P. Head,** M.D., Professor of Laryngology and Rhinology in the Chicago Post-Graduate Medical School. Vol. I. General Medicine, edited by **Frank Billings** and **J. H. Salisbury.** Chicago: The Year Book Publishers. 1906. (Price, $1.25) Vol. II. General Surgery, edited by **John B. Murphy.** (Price, $2.00; entire series, $10.00).

I. This number is taken up with the consideration of diseases of the respiratory organs, diseases of the circulatory organs, diseases of the blood and bloodmaking organs, general infectious diseases, metabolic diseases, diseases of the ductless glands, rheumatoid diseases, and diseases of the kidneys. Something over one hundred pages are set apart to the tuberculosis problem, which is one of the best condensations on that subject we have seen. The tent and other fresh air illustrations are instructive and indicate progress in this form of managing the consumptive. The suggestion by Peters, that superanuated trolley cars can be utilised to advantage by tuberculosis sufferers, is a good one and should be put into practical use. The remainder of the book is replete with interest and information for the practical physician.

II. The second volume of the 1906 series, devoted to general surgery, is edited by John B. Murphy. One always finds something of great interest when Murphy prepares the text. The surgery of the year has not been more compactly or usefully put before the profession than in this book. Abdominal surgery in general, the surgery of the stomach and intestines in particular, hernia, the surgery of the rectum, of the kidney, of the gall-bladder,—in short, the surgery of all the organs of the abdomen and pelvis is reported with sufficient detail to afford ample information on each topic. The abstracts are made with rare discrimination and many of them are elaborately illustrated. It is by far the best digest of the surgery of the year that has been sent out to physicians, as we have already intimated, and deserves well of every practitioner who expects to be prepared to meet surgical conditions.

**A Textbook of Materia Medica, Therapeutics, and Pharmacology.** By **George F. Butler,** Ph.G., M.D., Associate Professor of Therapeutics in the College of Physicians and Surgeons, Chicago. Fifth edition, thoroughly revised by **Smith Ely Jelliffe,** M.D., Ph.D., Professor of Pharmacology and Instructor in Materia Medica and Therapeutics in Columbia University (College of Physicians and Surgeons), New York. Octavo of 694 pages, illustrated. Philadelphia and London: W. B. Saunders Company. 1906. (Cloth, $4.00 net; Half Morocco, $5.00 net).

Since the last revision of this work, advances along its lines have wrought changes that render necessary a remodelling of most of the old forms. Professor Jelliffe has incorporated in this edition whatever there remained of value in the former, and has taken in all the recent studies in physiological chemistry, pharmacology, and the newer materia medica. It being intended as a book for students and practitioners; rather than for experimental workers in pharmacology, everything relating to the practical side of the topics dealt with has been made to take first place, to the exclusion of much that was found either obsolete or of only secondary importance.

This not only has changed the general arrangement of the book, but has served to reduce its size without diminishing its value; on the other hand, it has materially increased its usefulness. As now presented it takes a place in the front rank of textbooks on materia medica, therapeutics, and pharmacology, and is likely to meet the favor of medical schools as a working guide for laboratory pupils.

---

**Diseases of the Eye.** A handbook of Ophthalmic Practice. By **G. E. DeSchweinitz,** M.D., Professor of Ophthalmology in the University of Pennsylvania. Fifth edition, revised and enlarged. Octavo of 894 pages, 313 text-cuts and 6 chromo-lithographic plates. Philadelphia and London: W. B. Saunders Company. 1906. (Cloth, $5.00 net; Half Morocco, $6.00 net).

The distinction this author has attained in the field of ophthalmology has served to make his treatise so popular that frequent editions are demanded. This fact enables him to keep the material fresh and abreast of the progress making in this branch of medical science. In the present edition new chapters have been added on the following subjects: *x*-ray treatment of epithelioma, xeroderma pigmentosum; purulent conjunctivitis of young girls; jequiritol and jequiritol serum; *x*-ray treatment of trachoma; infected marginal ulcer; keratitis punctata syphilitica; uveitis and its varieties; eye-ground lesions of hereditary syphilis; macular atrophy of the retina; Worth's amblyoscope; stovain, alypin; Motais's operation for ptosis; Kuhnt-Muller's operation for ectropion; Haab's method for foreign bodies; and Sweet's *x*-ray method of localising foreign bodies. Other chapters have been rewritten. The illustrations are numerous and effective as aids to a completer understanding of the text. It is one of the foremost books for a beginner, or for a practitioner who desires to take up the study of ophthalmology.

---

**The Examination of the Function of the Intestines by means of the Test-Diet.** Its application in Medical Practice and its Diagnostic and Therapeutic value. By **Prof. Dr. Adolf Schmidt,** Physician-in-chief of the City Hospital Friedrichstadt in Dresden. Authorised Translation from the latest German Edition, by **Charles D. Aaron,** M.D., Professor of Diseases of the Stomach and Intestines in the Detroit Post-Graduate School of Medicine; Clinical Professor of Gastro-enterology in the Detroit College of Medicine; Consulting Gastro-enterologist to Harper Hospital, etc. With a frontspiece plate in colors. Crown Octavo, 91 pages. F. A. Davis Company, Publishers, 1914-16 Cherry Street, Philadelphia. (Price: extra cloth, $1.00 net).

The object of this brochure is to set forth the result of investigations, extending through eight years, into the function of the intestine, with the desire to institute an examination that could be carried out in practice, similar to the usual examinations of the stomach contents. The author confesses that he has not quite succeeded in reaching his goal; nevertheless, he has accomplished much in that direction, and has presented material that cannot fail to be of service to every practitioner dealing with intestinal diseases.

Dr. Aaron has given us a translation that preserves the original meaning of the author and, besides, makes smooth reading,—two very important points in rendering the German into English.

---

**Progressive Medicine, Vol. VIII, No. 2; June 1906.** A Quarterly Digest of Advances, Discoveries and Improvements in the Medical and Surgical Sciences. Edited by **Hobart Amory Hare,** M.D., Professor of Therapeutics and Materia Medica in the Jefferson Medical College of Philadelphia. Octavo, 368 pages, 31 illustrations. Lea Brothers & Co., Philadelphia and New York. (Per annum, in four cloth-bound volumes, $9.00; in paper binding, $6.00).

This number, which constitutes the second one of the eighth volume of the excellent periodical called Progressive Medicine, contains articles contributed by several men of conspicuous prominence in the medical profession. The titles are, hernia, by William B. Coley; surgery of the abdomen exclusive of hernia, by Edward Milton Foote; gynecology, by John G. Clark; diseases of the blood,—diathetic and metabolic diseases,—diseases of the spleen, thyroid gland, and lymphatic system, by Alfred Stengel; and ophthalmology, by Edward Jackson. Dr. Coley's article is well illustrated and Dr. Foote's is fairly so, but the others are deficient in this respect. However, the text is admirable and makes this number among the best yet issued.

---

**Transactions of the American Gynecological Society.** Volume 30, for the year 1905. **J. Riddle Goffe,** M.D., secretary. Philadelphia: William J. Dornan, printer. 1905.

The distinguishing feature of the meeting which created the material for this volume is that it was held at Niagara Falls. It is surprising that so much hard work could be done as was actually accomplished in that city of rest, and in sight of one of nature's great wonders.

The president, Dr. E. C. Dudley, of Chicago, in the introductory remarks to his address, took occasion to remind the Fellows, and indirectly the professional world, that gynecology is not dead as a specialty, that it has not "passed," and that the general surgeon has not absorbed it. The body of the address was taken up with a discussion of a phase of plastic surgery,—the incontinence of urine in women. Dr. Dudley described an operation which he devised for the relief of that condition, and which is admirably illustrated.

The entire volume is replete with interesting material, and is a credit to this excellent society.

**Nursing in the Acute Infectious Fevers.** By **George B. Paul**, M.D., Assistant Visiting Physician and Adjunct Radiographer to the Samaritan Hospital, Troy, N. Y. 12mo. volume of 200 pages, illustrated. Philadelphia and London: W. B. Saunders and Company. 1906. (Price, $1.50 net).

In this monograph will be found the essentials pertaining to nursing fever patients; indeed, it may be said that it reduces that form of nursing to a specialty. The author divides his work into three parts. The first treats of fevers in general; the second of each fever individually; the third deals with practical procedures and information necessary to the proper management of the various diseases discussed, such as antitoxins, bacteria, urine examination, poisons and their antidotes, enemata, topical applications, antiseptics, weights and measures, diet, and various other requisites pertaining to this special form of nursing. A table of the signs of the onset of the toxic effects of drugs, and one of poisons and their antidotes, add useful features to the book, which is one that every nurse should obtain and study.

**The World's Anatomists.** Concise Biographies of Anatomic Masters from 300 B. C. to the present time, whose names have adorned the literature of the medical profession. By **G. W. H. Kemper**, M.D., Professor of the History of Medicine in the Medical College of Indiana at Indianapolis. Revised and enlarged from the original serial publication in the Medical Book News. With eleven illustrations. Philadelphia: P. Blakiston's Son & Company. 1905.

The memoranda contained in this small brochure should attract the attention of every physician interested in the classics of medicine. They serve to refresh the memories of those who may have forgotten specific data, and to inform others who may not have learned them as yet. The author has done the profession a marked favor in collecting short sketches of the world's famous anatomists.

